# Microstructural and Mössbauer properties of low temperature synthesized Ni-Cd-Al ferrite nanoparticles

**DOI:** 10.1186/1556-276X-6-499

**Published:** 2011-08-18

**Authors:** Khalid Mujasam Batoo

**Affiliations:** 1 King Abdullah Institute for Nanotechnology, King Saud University, Riyadh, 11451, Saudi Arabia

**Keywords:** Nanoparticles, ferrites, SEM, TEM, IR spectroscopy, Mössbauer spectroscopy

## Abstract

We report the influence of Al^3+ ^doping on the microstructural and Mössbauer properties of ferrite nanoparticles of basic composition Ni_0.2_Cd_0.3_Fe_2.5 - *x*_Al*_x_*O_4 _(0.0 ≤ *x *≤ 0.5) prepared through simple sol-gel method. X-ray diffraction (XRD), scanning electron microscopy (SEM), energy dispersive X-ray, transmission electron microscopy (TEM), Fourier transformation infrared (FTIR), and Mössbauer spectroscopy techniques were used to investigate the structural, chemical, and Mössbauer properties of the grown nanoparticles. XRD results confirm that all the samples are single-phase cubic spinel in structure excluding the presence of any secondary phase corresponding to any structure. SEM micrographs show the synthesized nanoparticles are agglomerated but spherical in shape. The average crystallite size of the grown nanoparticles was calculated through Scherrer formula and confirmed by TEM and was found between 2 and 8 nm (± 1). FTIR results show the presence of two vibrational bands corresponding to tetrahedral and octahedral sites. Mössbauer spectroscopy shows that all the samples exhibit superparamagnetism, and the quadrupole interaction increases with the substitution of Al^3+ ^ions.

## Introduction

Nanoparticles of spinel ferrites have attracted great interest for a long time in fundamental science, especially in addressing the fundamental relationships between magnetic properties and their crystal chemistry and structure. Since nanoparticles have often novel properties that are different from their bulk properties due to their small size, they are becoming a core component of advanced materials that have wide practical applications with noble optical, electrical, magnetic, and catalytic properties [1,2]. Superparamagnetism is a unique feature of magnetic nanoparticles and is crucially related to many modern technologies, including ferrofluid technology [3], magnetic refrigeration [4], etc.

Ferrites are ferrimagnetic oxides, crystallizes into two magnetic sub-lattices, tetrahedral (A) site and octahedral (B) site. The electrical and magnetic properties, upon which their application depends, depend upon the cation distribution among these two sites. Ferrites are high-resistivity materials with low eddy current losses which make them potential materials for high-frequency applications such as microwave devices. The electrical resistivity of ferrites has been normally found to increase on doping or substituting with other oxides [5].

Several novel and non-equilibrium processing methods such as rapid solidification from the liquid state, mechanical alloying, plasma processing, vapor deposition, etc. have been developed during the past few decades to convert the microcrystalline materials to nanocrystalline materials in order to improve the physical and mechanical properties of the existing materials [6]. For example, magnetic behavior as a physical property is optimum in the nanocrystalline materials relative to conventional materials. It is well-known that the microstructure, especially the crystallite size, essentially determines the hysteresis loop of the soft ferromagnetic materials [7]. In the last two decades, various mechanical routes for producing ferrite magnetic powders (ferrites and metallic alloys) were introduced [7]. Mechanical alloying is one of the routine processes or preparation route of nanocrystalline structures by utilizing high-energy ball milling of materials to achieve alloys or composite materials with desired microstructures [8-10].

Ni-Cd ferrite, is a soft magnetic material, with a spinel crystal structure with widespread applications in recording heads, antenna rods, loading coils, microwave devices, core material for power transformers due to their high resistivity and low eddy current losses [11-13]. Nanocrystalline soft ferrites exhibit high coercivities and low saturation magnetization compared to the other conventional ferrites [14].

Cadmium is known to show strong preference for (A) sites in spinel ferrites. Consequently, CdFe_2_O_4 _is a normal spinel. On the other hand, NiFe_2_O_4 _is an inverse spinel where Ni^2+ ^and Fe^3+ ^occupy the octahedral and tetrahedral sites, respectively. With the mixing of Ni^2+ ^with Cd^2+ ^to form Ni-Cd ferrite, some of Fe^3+ ^ions migrate to octahedral positions and complexes of Fe^3+^/Cd^2+ ^reside in tetrahedral sites and Fe^3+^/Ni^2+ ^reside in octahedral sites [15-17]. Many reports on the synthesization of Ni-Cd ferrites are limited to ceramic techniques or solid state reaction methods [18-24]. To the best of our search we did not found any report on the synthesization of Ni-Cd ferrite nanoparticles though chemical route method. Among the various chemical route methods known, such as co-precipitation [25], sol-gel auto combustion [26], sol-gel [27], citrate-gel precursor [28] polymer pyrolysis [29], microemulsion [30], egg white [31], solvothermal method [32], hydrothermal [33], reverse micelle [34], the sol-gel method allows good control over the size of the material particles, which in turn decides their structural and transport properties (electrical and magnetic). The advantage of this method includes processing at low temperature, mixing at molecular level and fabrication of novel materials.

In the present work, we report the influence of f Al^3+ ^doping and grain size over microstructural, and Mossbauer properties of Ni_0.2_Cd_0.3_Fe_2.5 - *x*_Al*_x_*O_4 _ferrite nanoparticles using X-ray diffraction (XRD), scanning electron microscopy (SEM), transmission electron microscopy (TEM), energy dispersive X-ray (EDX), Fourier transformation infrared (FTIR), and Mössbauer spectroscopy techniques.

## Experimental details

### Preparation of the samples

Ferrite nanoparticles with chemical formula Ni_0.2_Cd_0.3_Fe_2.5 - *x*_Al*_x_*O_4 _(0.0 ≤ *x *≤ 0.5) were prepared through sol-gel method, using analytical grade chemicals: Ni(NO_3_)_2_.6H_2_O, Cd(NO_3_)_2_.4H_2_O, Al(NO_3_)_3_.9H_2_O, and Fe(NO_3_)_2_.9H_2_O as starting materials. Stoichiometric mixtures of the abovementioned materials were dissolved in deionized water and few drops of ethyl alcohol were added to it. Few drops of *N, N*-dimethylformamide C_3_H_7_NO (M.W.73.10) were added to the solution, to obtain the fine crystalline particles. The solution was allowed for gel formation on the magnetic stirrer at 75°C with constant stirring until gel was obtained. The gel formed was annealed at 90°C for 19 h followed by grinding for half an hour. The powder formed was heated for 36 h at 400°C to remove any organic material present and ground for half an hour [35].

### Measurements

PANanalytical X'Pert Pro X-ray diffractometer (PANalytical B.V., Almelo Netherland, instrument located at King Abdullah Institute for Nanotechnology, Riyadh, Saudi Arabia) with Cu Kα (*λ *= 1.54 Å) was used to study the single-phase nature and nanophase formation of the pure and doped Ni-Cd-Al ferrite nanoparticles at room temperature.

The microstructural analysis of the samples was carried out using a field emission scanning electron microscope (JSM 7600F, JEOL USA, Inc., instrument located at King Abdullah Institute for Nanotechnology, Riyadh, Saudi Arabia) and high-resolution transmission electron microscope (HRTEM) (Jeol 2010, JEOL USA, Inc., instrument located at King Abdullah Institute for Nanotechnology, Riyadh Saudi Arabia).

The IR measurements were carried out using Fourier transformation infrared spectrophotometer, Nicolet Impact 410 DSP (Nicolet Instrument Corp., instrument located at School of Nano and Materials Engineering, Changwon National University, South Korea) carried out in the range of 400 to 4,000 cm^-1^.

Mössbauer spectra of the nanoparticle samples were recorded at room temperature using Canberra series 30 multichannel analyzer with 25 mCi Co^57 ^source (Canberra Industries, Inc. Meriden, CT, USA). The calibration of the spectrometer was done using standard natural iron absorber.

## Results and discussion

### X-ray analysis

**Figure 1:**
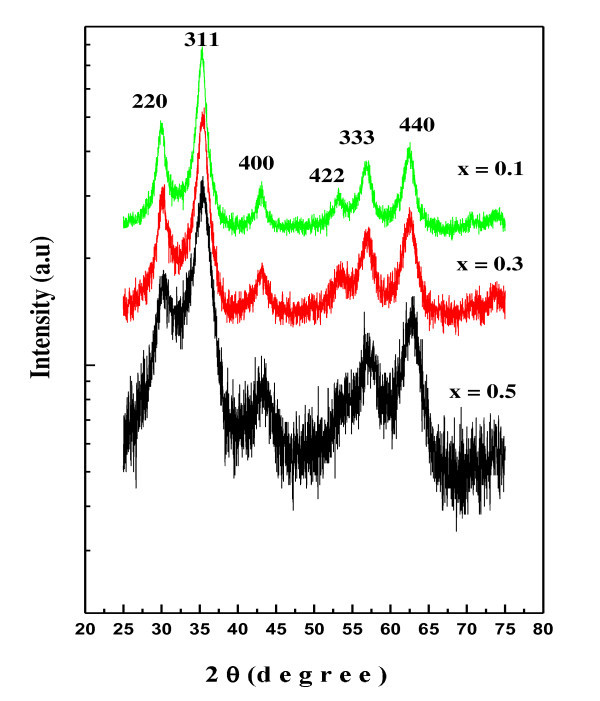
**X-ray diffraction pattern of Ni_0.2_Cd_0.3_Fe_2.5 - *x*_Al*_x_*O_4 _(0.0 ≤ *x *≤ 0.5) ferrite nanoparticles**.

The X-ray diffraction technique was employed for structural phase identification and magnetic nanoparticle formation of Ni_0.2_Cd_0.3_Fe_2.5 - *x*_Al*_x_*O_4 _(0 ≤ *x *≤ 0.5) ferrites. Figure 1 shows the powder X-ray diffraction pattern of Ni_0.2_Cd_0.3_Fe_2.5 - *x*_Al*_x_*O_4 _(0 ≤ *x *≤ 0.5) ferrites. The XRD pattern analyzed using Powder-X software (Institute of Physics, Chinese academy of siences, Beijing, People's Republic of China, software located at KingAbdullah Institute for Nanotechnology, Riyadh Saudi Arabia), confirmed single-phase cubic spinel structure formation with *Fd3m *space group. The most intense peaks in all specimens, indexed as (220), (311), (400), (422), (333), and (440) are found to match well with single-phase cubic spinel ferrites. The crystallite size was calculated from the XRD data using Scherrer formula:

(1) $$\Gamma =\frac{0.98\lambda }{{\left(L\right)}_{\text{vol}}\cos\theta },$$

where *Γ *is the average crystalline dimension perpendicular to the reflecting phases, *λ *the X-ray wavelength, *θ *the Bragg's angle, and (*L*)_vol _the volume-weighted average column length, i.e., the number of reflecting planes times their effective distance "d." For spherical particle (*L*)_vol _equals 0.75(*D*)_vol_, where *D *is the grain diameter. The average crystallite sizes of all the samples were determined using a (301) diffraction peak broadening technique and is found to be in the range of 3 nm to approximately 7 nm (± 1).

**Figure 2:**
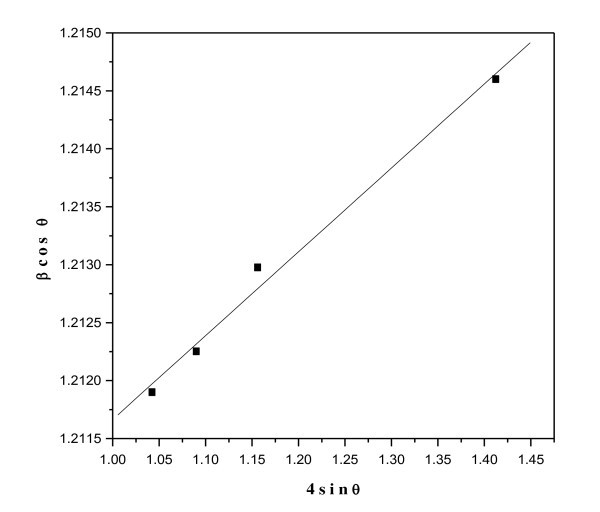
**Strain graph of the nanoparticles of Ni_0.2_Cd_0.3_Fe_2.5 - *x*_Al*_x_*O_4 _(0.0 ≤ *x *≤ 0.5) ferrite nanoparticles**.

Figure 2 shows the strain measurements for all the compositions of Ni_0.2_Cd_0.3_Fe_2.5 - *x*_Al*_x_*O_4 _(0 ≤ *x *≤ 0.5) ferrite nanoparticles. It is seen that the variation of 4 sin*θ *with *β*cos*θ *is linear for all the samples, which shows that the strain in the samples increases with the decreasing size of the nanoparticles.

### Scanning electron microscopy

**Figure 3:**
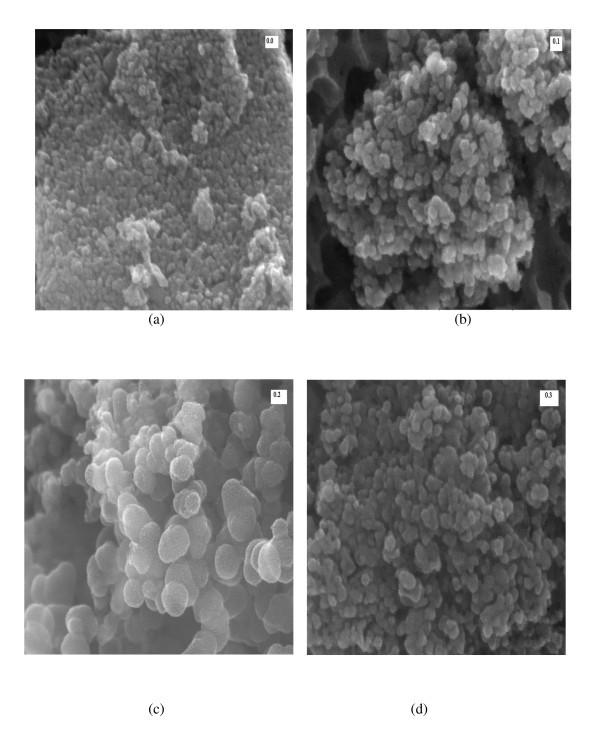
**SEM micrographs of Ni_0.2_Cd_0.3_Fe_2.5 - *x*_Al*_x_*O_4 _(0.0 ≤ *x *≤ 0.5) ferrite nanoparticles**.

In order to understand the morphology, grain size, and shape of the grown nanoparticles, SEM measurements were carried out. The SEM micrographs were taken at 1,000 magnifications by selecting different parts of the samples. The SEM images of pure and substituted samples are shown in Figure 3a, b, c and 3d. It is clear from the micrographs that the microstructure changes with the increasing concentration of Al^3+ ^ions. A closer look on these micrographs shows that grown nanoparticles are spherical in shape and have intergranular diffusion. Also, it is seen that the number of pores increases with the increasing doping concentration which results in lesser densification or more porosity.

### Energy dispersive X-ray

The chemical composition of samples was estimated by EDX technique. The EDX pattern confirms homogeneous mixing of Ni, Cd, Fe, Al, and O atoms in pure and doped samples. Table 1 presents the detailed estimated composition of Ni_0.2_Cd_0.3_Fe_2.5 - *x*_Al*_x_*O_4 _(0 ≤ *x *≤ 0.5) ferrite nanoparticles. The observed composition is almost equal to that of the samples produced by stoichiometric calculations while taking oxygen as balanced.

**Table 1:** The EDX parameters of Ni_0.2_Cd_0.3_Fe_2.5 - _*_x_*Al*_x_*O_4 _(0.0 ≤ *x *≤ 0.5) ferrite nanoparticles

Chemical composition (EDX)										
Composition	wt.%					at.%				
(*x*)	Ni	Cd	Fe	Al	O	Ni	Cd	Fe	Al	O
0.0	04.06	13.92	53.44	-	Balanced	02.35	04.22	32.59	-	Balanced
0.1	05.72	14.49	49.16	02.71	Balanced	03.30	04.37	29.82	-	Balanced
0.2	05.26	13.70	47.70	03.68	Balanced	02.93	03.99	27.95	-	Balanced
0.3	05.18	13.20	47.26	05.15	Balanced	02.88	03.83	27.58	-	Balanced

### High-resolution transmission electron microscopy

**Figure 4:**
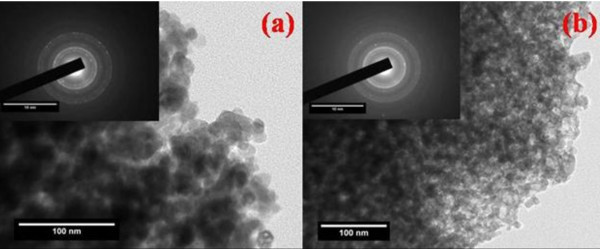
**TEM micrograph**. TEM micrograph for the composition *x *= 0.0 and 0.1 with inset showing their SAED micrographs.

The representative illustration of HRTEM micrographs of the synthesized nanoparticles along with the selected area electron diffraction (SAED) pattern for pure and doped Ni-Cu-Zn ferrite nanoparticles are presented in Figure 4. The micrographs show largely agglomerated nanoparticles of the sintered powder samples. An overview of the TEM image of nanoparticles shows that the particles have a size distribution of 2 to 8 nm (± 1 nm). Such aggregate formation and broader size distribution are characteristic of mechanically activated nanosized particles. The agglomeration of particles may be because they experience a permanent magnetic moment proportional to their volume. Very few large particles having a size around 14 nm have also been found. It has been observed that the size of the particles obtained through HRTEM measurement corroborates well with crystallite size obtained from XRD analysis. The shape of the majority of the particles appears to be nonspherical. In the SAED image of synthesized nanoparticles, distinct rings confirming good crystallinity are clearly visible. The observed crystallographic "*d*" values of 2.52 Å correspond to the lattice space of (311) plane of the Ni-Cd-Al ferrite system. The observed crystallographic "*d*" values agree well with those obtained from XRD analysis. The results of XRD and HRTEM study divulge that all the samples are well crystalline nanosized spinel ferrites. The average particle diameter was found to be 5 nm which is in good agreement with the XRD results.

**Figure 5:**
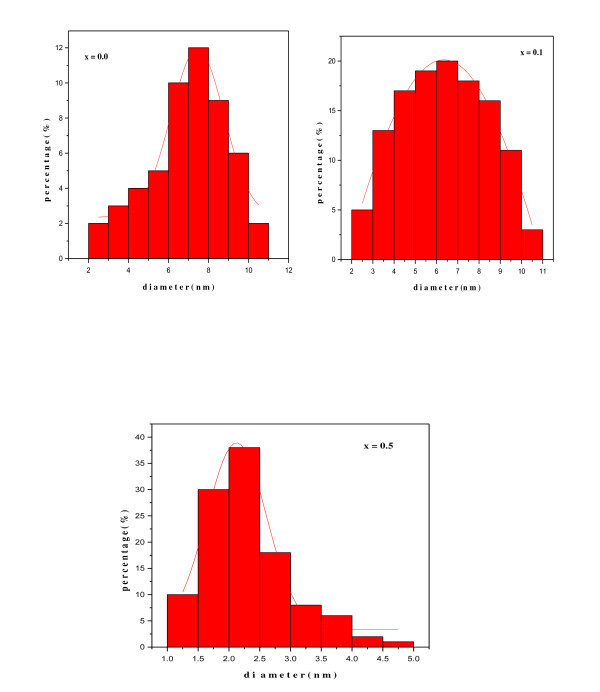
**Typical histograms showing size distribution of Ni_0.2_Cd_0.3_Fe_2.5 - *x*_Al*_x_*O_4 _(0.0 ≤ *x *≤ 0.5) ferrite nanoparticles**.

Figure 5a, b, c shows the typical histograms of size distribution for Ni_0.2_Cd_0.3_Fe_2.5 - *x*_Al*_x_*O_4 _nanoparticles with a diameter of 2, 8, and 7 nm, respectively, indicating the quality of spherical Ni_0.2_Cd_0.3_Fe_2.5 - *x*_Al*_x_*O_4 _nanoparticles are very high in terms of size distribution.

### Fourier transformation infrared spectroscopy

**Figure 6:**
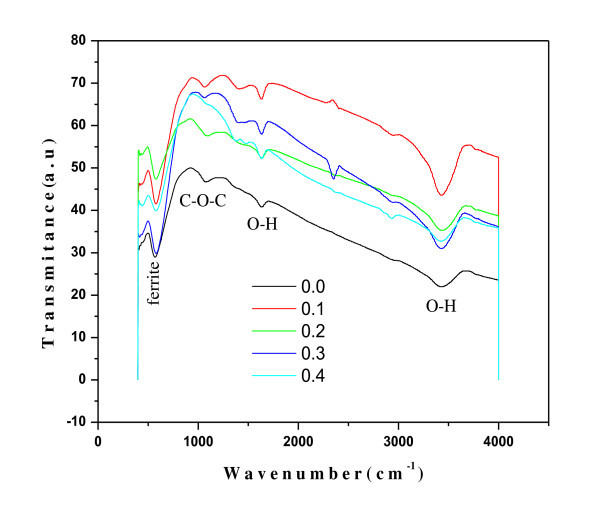
**FTIR spectra of Ni_0.2_Cd_0.3_Fe_2.5 - *x*_Al*_x_*O_4 _(0.0 ≤ *x *≤ 0.5) ferrite nanoparticles**.

The infrared spectroscopy gives information about the chemical and molecular structure changes in ferrites due to the changes in Fe-O bond during heat treatment or when some foreign atom is introduced in the parent ferrite compound. Figure 6 shows the FTIR spectra of grown Al doped Ni-Cd ferrite nanoparticles in the range of 400 to 4,000 cm^-1^. For ferrites, generally two assigned absorption bands appear around 600 cm^-1^: *ν*_1_, which is attributed to stretching vibration of tetrahedral group Fe-O and that around 400 cm^-1^: *ν*_2_, which is attributed to the octahedral group complex Fe-O.

The two strong bands that appear around 579 cm^-1 ^and 420 cm^-1 ^are the characteristic bands of Ni_0.2_Cd_0.3_Fe_2.5 - *x*_Al*_x_*O_4 _ferrite revealing the formation of Ni-Cd-Al ferrite. The absorption band *ν*_1 _appears around 579 cm^-1 ^and the absorption band *ν*_2 _appears around 420 cm^-1^. The difference between *ν*_1 _and *ν*_2 _is due to the changes in bond length (Fe-O) at octahedral and tetrahedral sites [36]. The spectra also show a shift due to the introduction of Al^3+ ^ions. The tetrahedral site bands are shifted from lower band values to higher band values, i.e., from 574.32 to 579.21 cm^-1^, which is attributed to the stretching of Fe-O bonds on substitution of Al ions. The octahedral band sites on the contrary shift towards lower frequency region from 429.24 to 417.51 cm^-1 ^with Al addition, which is attributed to the shifting of Fe towards oxygen ion on occupation of octahedral sites by Al ions [37].

### Mossbauer spectroscopy

**Figure 7:**
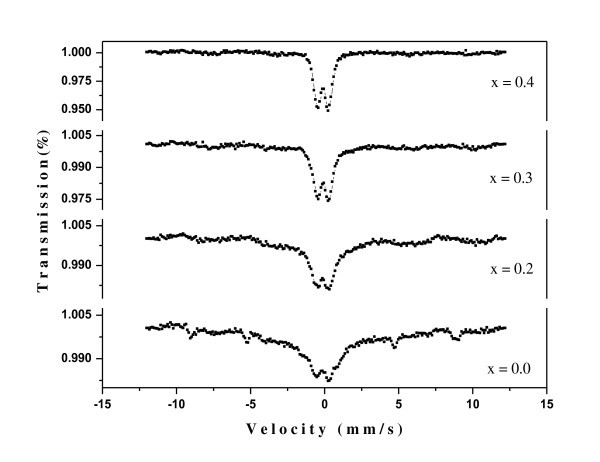
**Mössbauer pattern of Ni_0.2_Cd_0.3_Fe_2.5 - *x*_Al*_x_*O_4 _(0.0 ≤ *x *≤ 0.5) ferrite nanoparticles at 300 K**.

Mössbauer spectroscopy (MS) of the fabricated nanoparticles was recorded at room temperature (300 K) using Canberra series 30 Multichannel Analyzer and 25 mCi Co^57 ^source (Canberra Industries, Inc.). The calibration of the spectrometer was done using standard natural iron absorber. The values of the isomer shift, quadrupole splitting values are presented in Table 2. The observed isomer shift values were calculated with reference to α-Fe at 300 K and are consistent with the literature reports [38,39]. The presence of doublet indicates the characteristic of the paramagnetic behavior of Ni-Cd-Al ferrite, as shown in Figure 7. A doublet arises from superparamagnetic nanoparticles that relax at a faster rate than the MS measurement time (10^-9 ^s). A significant change in the isomer shift of Ni_0.2_Cd_0.3_Fe_2.5_Al*_x_*O_4 _is observed with progressive doping of Al^3+ ^ions, which indicate that the S-electron charge distribution of Fe^3+ ^ion is influenced by Al substitution. The samples *x *= 0.0 (8 nm) and 0.2 (5 nm) are superparamagnetically relaxed, and the relaxation in these samples decreases while intensity of the paramagnetic doublet increases with the decreasing size of the particle or Al^3+ ^doping, which may be due to the interaction of the electric field gradient (EFG) with the quadrupole moment of Fe^57 ^nucleus and the reduction of interaction between Fe ions due to dilution of B sublattice by Al^3+ ^ions. The analysis of the data shows that quadrupole splitting increases with progressive doping, which means the interaction of EFG with the quadrupole moment of Fe^57 ^nucleus increases. In other words, the interaction of EFG with the quadrupole moment of Fe^57 ^is enhanced and the hyperfine interaction goes zero with reducing grain size [40]. The quadrupole doublet pattern clearly shows that all the samples exhibit superparamagnetism. The results obtained are consistent with the results of the vibrational sample magnetometer and are in well agreement with those reported earlier in the literature [41-43]. The presence of paramagnetic doublet may be attributed to the small particle size. It is seen that the isomer shift values of the A site are less than those of the B site. This conclusion has been proven by many authors [44-47]. The values of Δ*E *indicate the degree of deviation from cubic symmetrical structure. The absolute values of Δ*E *increases with the decreasing particle size, and the asymmetrical electric fields surrounding the Mössbauer nucleus will be strengthened [39]. As the particle sizes are small, the crystallization will be incomplete. The decrease in hyperfine field with the doping may be also explained on the basis that Al^3+ ^ions prefer to occupy the B site. The introduction of some nonmagnetic Al^3+ ^ions decreases the Fe number at B site which in turn, weakens the intersublattice (AB) interactions between Fe ions.

**Table 2:** Mössbauer parameter of the Ni_0.2_Cd_0.3_Fe_2.5 - _*_x_*Al*_x_*O_4 _ferrite nanoparticles at room temperature

(*x*)	Grain size		Paramagnetic doublet
	D (nm)	Isomer shift *δ *(mm/s)	Quadrupole splitting Δ*E *(mm/s)
*x *= 0.0	8	0.22	0.49
*x *= 0.2	5	0.23	0.51
*x *= 0.3	4	0.33	0.54
*x *= 0.4	2	0.37	0.55
Error		± 0.02	± 0.02

## Conclusions

Nanoparticles of Ni_0.2_Cd_0.3_Fe_2.5_Al*_x_*O_4 _ferrites were synthesized through the sol-gel method. The FTIR results show the presence of two vibrational modes corresponding to tetrahedral and octahedral sites. Mössbauer spectroscopy results confirm that all the samples exhibit superparamagnetism. The samples show the presence of paramagnetic doublet due to quadrupole interaction. The intensity of the paramagnetic doublet increases with increasing concentration of Al^3+ ^ions or with decreasing particle size.

## Competing interests

The author declares that they have no competing interests.
